# Does Curative Radiotherapy Impact Fatigue Onset in Prostate Cancer Patients? An Analysis of 1253 Patients

**DOI:** 10.1002/cnr2.70525

**Published:** 2026-03-27

**Authors:** Milena Ferro, Savino Cilla, Gabriella Macchia, Giuseppe Limosani, Paolo Bonome, Gianpiero Mastrogiorgio, Vincenzo Picardi, Carmela Romano, Marica Ferro, Mariangela Boccardi, Donato Pezzulla, Milly Buwenge, Alessio G. Morganti, Francesco Deodato

**Affiliations:** ^1^ Radiation Oncology Unit, Responsible Research Hospital Campobasso Italy; ^2^ Medical Physics Unit, Responsible Research Hospital Campobasso Italy; ^3^ Radiation Oncology, IRCCS Azienda Ospedaliero‐Universitaria di Bologna Bologna Italy; ^4^ Department of Experimental, Diagnostic, and Specialty Medicine – DIMES Alma Mater Studiorum, Bologna University Bologna Italy; ^5^ Istituto di Radiologia, Università Cattolica del Sacro Cuore Rome Italy

**Keywords:** cancer‐related fatigue, CLAS, prostate cancer, radiotherapy

## Abstract

**Background:**

This study investigates the impact of radiotherapy (RT) on fatigue levels in prostate cancer (PC) patients, considering the significant physical and psychological impact of fatigue associated with cancer and its treatments.

**Methods:**

PC patients undergoing a radical treatment from December 2002 to September 2022 were retrospectively evaluated. Fatigue was assessed using the Cancer Linear Analogue Scale (CLAS) across three dimensions: wellbeing (CLAS1), energy level (CLAS2), and daily activity performance (CLAS3), measured at baseline (T0), 1 month (T1), and 12 months (T2) post‐RT. Changes in CLAS scores ≥ 2 points from T0 were deemed clinically significant for RT‐induced fatigue.

**Results:**

The cohort consisted of 1253 patients, with a median age of 72 years (range 45–90). Approximately 30% of patients experienced moderate or high levels of fatigue at baseline. At T1, RT‐related fatigue onset (decrease of CLAS1, 2, and 3 values) was observed in 10.8%, 14.3%, and 14.8% of patients, respectively. These figures slightly increased at T2 (12.7%, 18.8%, and 19.4%, respectively). Logistic regression identified hypofractionated RT, ADT, surgery, alcohol consumption, and higher‐grade toxicities as predictors of worsened fatigue across various dimensions.

**Conclusion:**

In this extensive cohort of PC patients, approximately 30% experienced moderate to severe fatigue before initiating RT, with less than 20% reporting new or exacerbated fatigue post‐treatment. Factors including treatment‐related toxicities, hypofractionation, alcohol use, and ADT were significant contributors to fatigue. These findings underscore the complexity of managing fatigue in PC, highlighting the influence of both treatment modalities and lifestyle factors.

## 1 Introduction

Fatigue is a common symptom reported by cancer patients at all stages of the disease. Its prevalence ranges from 62% to 85% during active treatment, potentially reaching 100%, and slightly decreases to about 50% in the years following treatment [1, 2]. The causes of fatigue are not fully understood; however, it is believed to result from a complex interplay of physiological, clinical, and psychological factors [3, 4]. A significant contributor to this condition appears to be the dysregulation of pro‐inflammatory cytokines, which may begin at diagnosis, originate from the tumor, or result from tissue damage caused by chemotherapy and radiotherapy (RT) [5, 6]. Numerous studies have explored the prevalence of fatigue in patients undergoing RT for various types of cancer [7, 8, 9, 10].

In prostate cancer (PC) treatment, standard fractionation (1.8–2 Gy) has long been the predominant RT schedule. Recent advances have shown that moderate‐ and ultra‐hypofractionation are more beneficial from a radiobiological perspective [11]. Furthermore, the adoption of advanced techniques such as Volumetric Modulated Arc Therapy (VMAT) and Intensity Modulated RT (IMRT) has enabled the administration of high doses per fraction while preserving normal tissue tolerance and minimizing side effects [12, 13, 14]. This reduction in potential toxicity may also alleviate fatigue.

Fatigue in PC patients has been extensively studied, revealing a broad prevalence and various findings regarding its correlation with treatment factors, such as RT dose and volumes, and its association with other treatment modalities [15, 16, 17, 18]. Leveraging our extensive experience in PC treatment [19, 20, 21, 22, 23], we aimed to examine the impact of RT on patient fatigue.

## 2 Material and Methods

### 2.1 Study Design and Setting

This retrospective, single‐center observational cohort study included 1253 consecutive prostate cancer (PC) patients treated with curative‐intent radiotherapy (RT) at the Radiation Oncology Unit of the Campobasso Responsible Research Hospital between December 2002 and September 2022.

All treatments were administered in accordance with institutional protocols and current clinical guidelines. No additional diagnostic procedures, experimental interventions, or deviations from standard clinical management were performed for research purposes. Clinical and treatment‐related data were prospectively collected in an institutional database as part of routine clinical practice and subsequently retrospectively analyzed for this study.

The principal objective was to evaluate fatigue variation following radiotherapy. Secondary objectives included the identification of clinical and treatment‐related variables associated with fatigue worsening.

### 2.2 Inclusion Criteria and Treatment

Eligible patients were ≥ 18 years old, had histologically confirmed PC diagnosed by transrectal ultrasound‐guided or MRI–US fusion biopsy, and were treated with definitive or postoperative radiotherapy. Patients previously irradiated were excluded. Patients from all risk categories (very low, low, favorable, and unfavorable intermediate, high, and very high risk) were included. Radiotherapy was delivered using Elekta Precise and Versa HD linear accelerators with VMAT or IMRT techniques. Radiotherapy was delivered with curative intent (definitive, adjuvant, or salvage) using standard, hypofractionated, or stereotactic schedules based on clinical indication. Target volumes included the prostate gland, prostate bed, and, optionally, pelvic lymph nodes. Androgen deprivation therapy (ADT) was administered in neoadjuvant, concomitant, and/or adjuvant settings when clinically indicated.

### 2.3 Fatigue Assessment and Endpoints

Fatigue was assessed using the Cancer Linear Analogue Scale (CLAS), which evaluates three domains: wellbeing (CLAS1), energy level (CLAS2), and ability to perform daily activities (CLAS3), each scored from 0 to 10, with higher scores indicating lower fatigue. Assessments were performed at baseline before radiotherapy (T0), 1 month after treatment (T1), and 12 months after treatment (T2). A trained nurse administered and recorded the assessments. Fatigue levels were categorized as high (0–3.9), moderate (4–6.9), and low (7–10). Clinically significant variation was defined as a ≥ 2 point change from baseline, based on previously established consensus thresholds. The primary endpoint was clinically significant fatigue worsening at T1 and T2. Secondary endpoints included correlations between fatigue variation and clinicopathological or treatment‐related variables.

### 2.4 Toxicity and Variables

Acute and late toxicities were graded according to RTOG/EORTC criteria [24]. Variables analyzed included age, comorbidities, ECOG performance status, smoking and alcohol habits, ADT use and duration, surgical history, fractionation schedule, prophylactic nodal irradiation (PNI), and acute and late toxicities.

### 2.5 Statistical Analysis

Baseline characteristics were summarized using descriptive statistics. Comparisons among fatigue categories were performed using the Kruskal‐Wallis test. Logistic regression analyses explored associations between fatigue changes and clinical or treatment‐related factors. Statistical analyses were conducted using XLSTAT (Addinsoft, NY, USA).

### 2.6 Ethical Considerations

The study was conducted in accordance with the principles of the Declaration of Helsinki. The retrospective analysis of fully anonymized data collected during routine clinical practice was deemed consistent with the ongoing research activities of our institution and did not involve any research‐driven intervention or modification of standard care. The study protocol was reviewed and approved by the Internal Institutional Scientific Board during its session on November 12, 2025 (Protocol number 12112025). All patients had previously provided written informed consent.

## 3 Results

Between December 2002 and September 2022, 1253 PC patients received treatment at the Radiotherapy unit of the Campobasso Responsible Research Hospital. The median age was 72 years (range, 45–90). A significant proportion (66.7%) had zero or one comorbidity, and 96% demonstrated an optimal or good performance status (ECOG: 0–1). RT was the exclusive treatment for 61.4% of patients, with 84.3% receiving ADT. In about one third of patients (32.9%), the length of ADT was 24 months, whilst the duration of ADT was 6–12 months in 39.5% of patients. The majority (86.3%) were treated using moderate‐ or ultra‐hypo‐fractionated schedules, while the rest (13.7%) followed standard schedules. Table 1 summarizes patient characteristics and treatment factors.

**TABLE 1:** Patient characteristics and treatment factors.

	*N* (%)
Patients with CLAS evaluation	1214 (97)
Median age (range)	72 (45–90)
Risk class	
VLR	12 (1)
LR	57 (4.7)
FIR	252 (20.8)
UIR	118 (9.7)
HR	556 (45.8)
VHR	219 (18)
Incorrect habits	
Smoke	539 (44.4)
Alcohol	770 (63.4)
ECOG	
0–1	1166 (96)
2–3	48 (4)
Comorbidities	
0–1	810 (66.7)
2–6	404 (33.3)
RT treatment intent	
Curative	745 (61.4)
Adjuvant	279 (23)
Salvage	190 (15.6)
RT fractionation	
Standard fractionation	74 (6.1)
Hypofractionated RT	974 (80.2)
Stereotactic RT	166 (13.7)
Pelvic lymph nodes RT	
Yes	1085 (89.4)
No	129 (10.6)
ADT	
Yes	1023 (84.3)
No	191 (15.7)

Abbreviations: ADT: androgen‐deprivation therapy; FIR: favorable intermediate risk; HR: high risk; LR: low risk; UIR: unfavorable intermediate risk; VHR: very high risk; VLR: very low risk.

A total of 1214 patients (97%) completed the Cancer Linear Analogue Scale (CLAS) evaluation at baseline (T0), 1 month (T1), and 12 months after RT (T2). Grade 1–2 (G1‐2) toxicities, predominantly genitourinary (GU) disorders, were reported by 74% of patients. Grade 4 (G4) GU toxicities were reported by 5 patients (< 1%), characterized by acute bladder obstruction immediately post‐RT. Grade 3 (G3) toxicities were reported by 17 and 10 patients for GU and gastrointestinal (GI) issues, respectively, with an additional three patients experiencing G3 hematologic toxicity. Late toxicities included G3 issues in eight patients (0.6%), mainly due to rectal bleeding requiring intervention, and G1–G2 toxicities in 441 patients (36.3%), primarily proctitis and moderate GU frequency.

**FIGURE 1:**
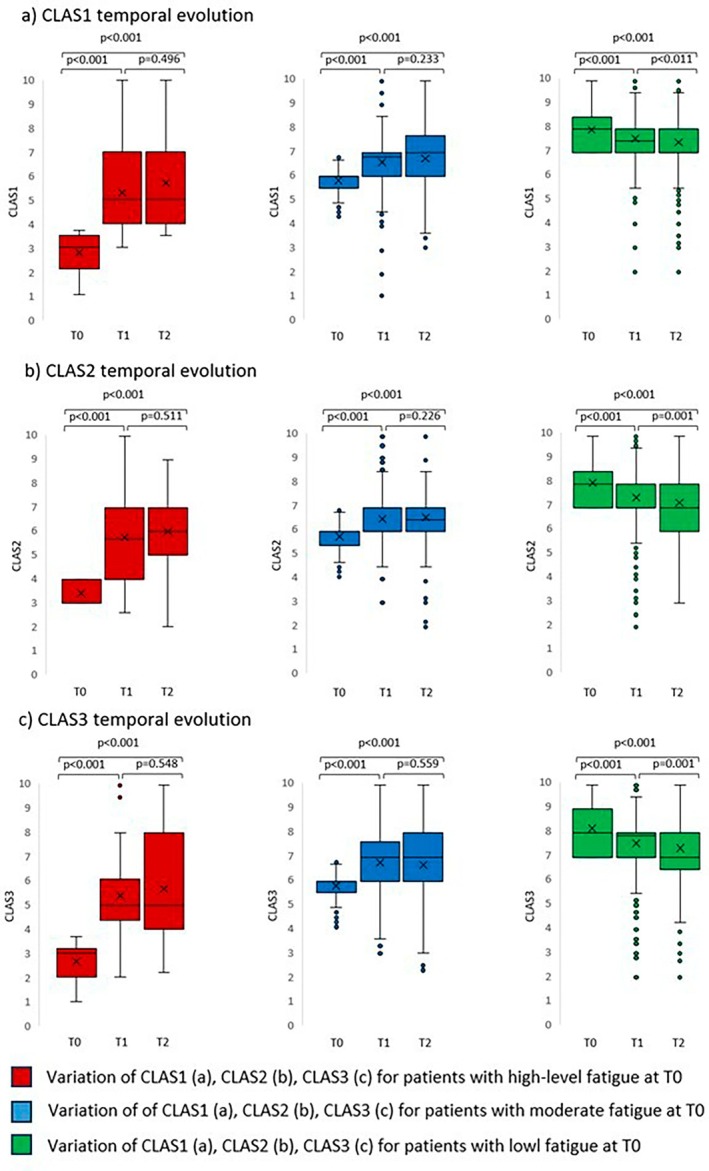
CLAS value at T0, T1, and T2 for all patients stratified by fatigue level. Red, blue, and green box‐plots refer to patients with high, moderate, and low fatigue level, respectively. P values were calculated using Kruskal–Wallis test. CLAS 1: Well‐being; CLAS 2: Energy level; CLAS 3: Ability to perform daily activities; T0: Baseline; T1: 1 month after RT; T2: 12 months after RT.

The median CLAS scores across all domains were 7 (range 0–10) at T0, remaining stable at T1 and T2, with a slight increase to 7.2 for CLAS1. The calculated standard deviation (SD) of CLAS values consistently ranged between 1.8 and 2.2 across all classes. Figure 1 displays the fatigue level distribution across different timepoints, showing that approximately 30% of patients experienced moderate or high fatigue levels at baseline. This remained stable for CLAS1 but showed a slight increase at T1 (35.3% for CLAS2 and 30.9% for CLAS3) and T2 (38.9% and 33.5% for CLAS2 and CLAS3, respectively).

**FIGURE 2:**
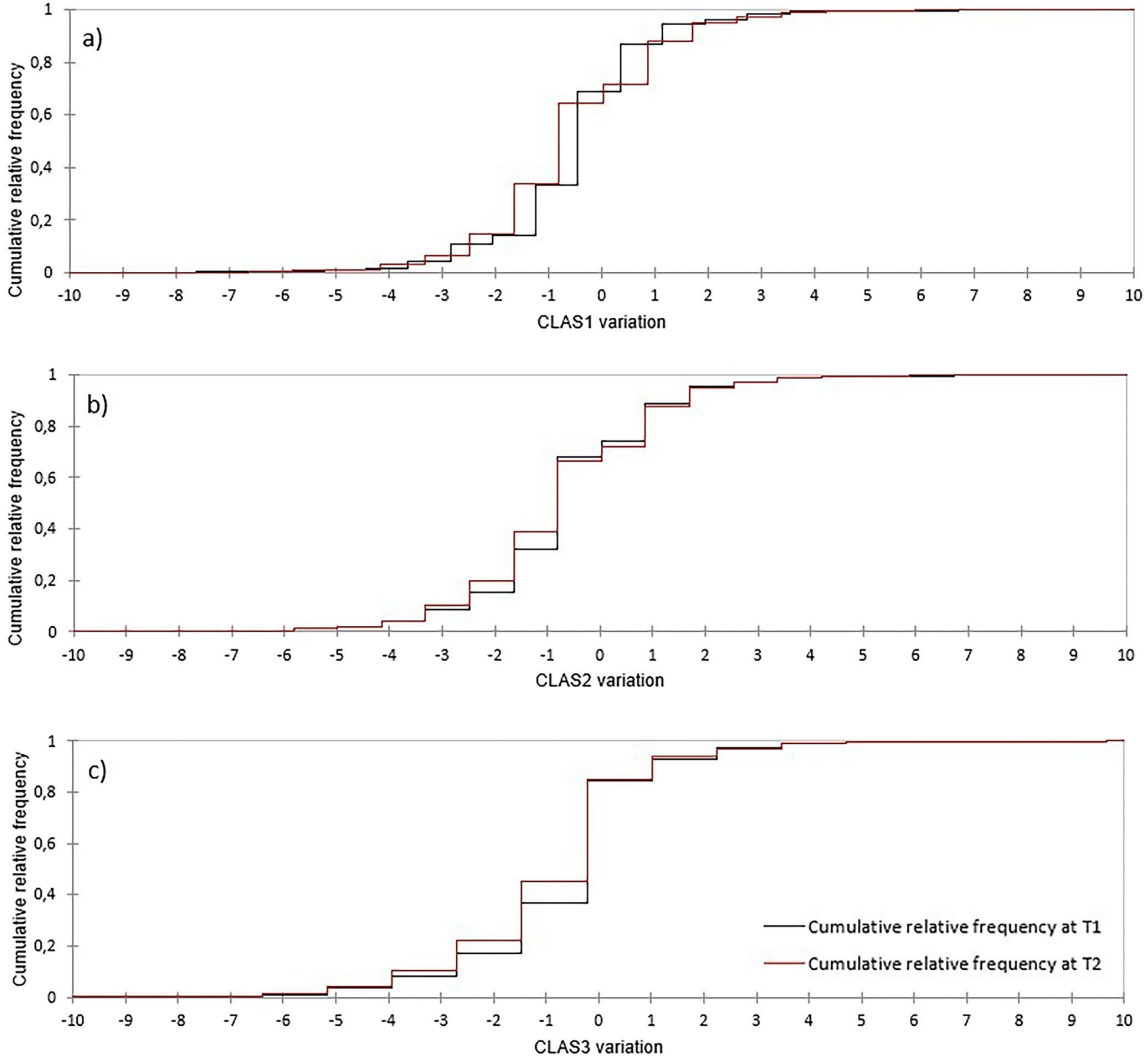
Cumulative relative frequency of CLAS variation at T1 and T2 for (a) CLAS1, (b) CLAS2, and (c) CLAS3 scores. The proportion of patients exceeding the ≥ 2 point threshold is represented by the area < −2. CLAS 1: Wellbeing; CLAS 2: Energy level; CLAS 3: Ability to perform daily activities.

RT‐related fatigue onset, defined as a decrease in CLAS scores, was observed in 10.8%, 14.3%, and 14.8% of patients at T1, and 12.7%, 18.8%, and 19.4% at T2 for CLAS1, CLAS2, and CLAS3, respectively. Figure 2 shows the cumulative relative frequency of CLAS variation at different timepoints.

Logistic regression analysis showed significant correlations between the worsening of patient wellbeing (CLAS1) one year post‐RT and both hypo‐fractionated RT (*p* = 0.004) and late toxicities higher than grade 1 (*p* = 0.017). Although the association between ADT and the worsening of wellbeing showed a statistical trend, it did not reach conventional levels of significance (*p* = 0.056). Similarly, hypo‐fractionated RT and surgery were associated with decreased energy levels (CLAS2) at T1 (*p* = 0.023 and *p* = 0.033, respectively), as were grade 1 or higher GU acute toxicities (*p* = 0.016) at T1, and GI acute toxicities at T2 (*p* = 0.045). Hypofractionated RT and alcohol consumption were linked to reduced ability to perform daily activities (CLAS3) at T1 (*p* = 0.014 and *p* = 0.010, respectively). Additionally, at T2, ADT (*p* = 0.010), alcohol consumption (*p* = 0.043), and late toxicities higher than G1 (*p* = 0.017) were correlated with deteriorations in CLAS3. Finally, surgery correlates with CLAS3 both at T1 (*p* = 0.001) and T2 (*p* = 0.004) (Table 2). No significant correlations were found between fatigue variation and factors such as age, PNI, comorbidities, and smoking habits, as well as between patients with fatigue at baseline and toxicity, except for CLAS1 and GU acute toxicity (*p* = 0.09; data not shown).

**TABLE 2:** Logistic regression of variables predicting the onset of RT‐related fatigue.

Variables	CLAS1		CLAS2		CLAS3	
	1 month [T1]	1 year [T2]	1 month [T1]	1 year [T2]	1 month [T1]	1 year [T2]
Age	—	—	—	—	—	—
Hypofractionation	—	0.004	0.023	—	0.014	—
Surgery	—	—	0.033	—	0.001	0.004
Pelvic Nodal irradiation	—	—	—	—	—	—
Comorbidities	—	—	—	—	—	—
GU acute toxicity > G1	—	—	0.016	—	—	—
GI acute toxicity > G1	—	—	—	0.045	—	—
ADT	—	0.056	—	—	—	0.010
Smoking habit	—	—	—	—	—	—
Alcohol consumption	—	—	—	—	0.010	0.043
Late toxicity > G1	—	0.017	—	—	—	0.017

Abbreviations: ADT: Androgen‐Deprivation Therapy; CLAS 1: well‐being; CLAS 2: energy level; CLAS 3: ability to perform daily activities; G: grade; GI: gastro‐intestinal; GU: genito‐urinary; T0: baseline; T1:1 month after RT; T2:12 months after RT.

## 4 Discussion

This study aimed to explore the impact of RT on fatigue in a large cohort of PC patients treated with advanced techniques over two decades at our institution. We found that RT is associated with an increase in fatigue levels in a minority of patients. The main factors correlated with fatigue, both at early and late stages post‐RT, were hypofractionation, ADT, and treatment‐related toxicities.

Supporting our findings, Joseph et al. [16] reported that 15%–30% of PC patients experienced a relevant increase in fatigue after RT, particularly with larger doses per fraction. Similarly, the PACE‐B trial [25] demonstrated that ultra‐hypofractionated RT led to increased fatigue compared to standard or moderate hypofractionation. These data are consistent with our observations of hypofractionation impact on fatigue across wellbeing, energy levels, and daily activities.

Although fatigue is often associated with cancer treatment, it may also result from the disease itself, even before any therapeutic intervention. Indeed, in this cohort one third of the population presented fatigue prior to RT. This early presentation is well described in the literature, and it seems to be a consistent predictor of post‐treatment fatigue [26]. Factors contributing to fatigue before treatment include demographic and medical factors, such as younger age, having children at home, and higher body mass index, as well as symptoms including depressed mood and sleep disturbance. Moreover, cancer‐related inflammation may contribute to fatigue even before treatment in vulnerable patients [27].

Contrary to the hypothesis proposed by Joseph et al. [16] that PNI might elevate fatigue levels due to larger irradiation volumes, our data did not support this association. This finding is consistent with those of Wallis et al. [28], who noted no significant differences in physical functioning or fatigue among PC patients receiving PNI. However, the low percentage of patients who did not have PNI suggests that the lack of correlation may be due to inadequate statistical power rather than a true lack of effect.

ADT emerged as a significant factor affecting fatigue 1‐year post‐RT, likely due to the reduction in serum testosterone and haemoglobin levels, alongside alterations in adrenal hormone production, including stress‐related corticoids [15, 29, 30, 31]. This correlation between ADT and increased fatigue has been documented previously, highlighting a notable difference in chronic fatigue prevalence between patients undergoing RT with or without ADT [32].

Concerning surgery, in this trial, an impact on daily activities both at 1 month and 1 year post‐treatment was found in logistic regressions, as well as per energy level at one month. Few studies have specifically investigated levels of fatigue in men treated exclusively with radical prostatectomy for PCa; however, in the literature relevant long‐lasting fatigue is reported by 14%–21% of men after surgery. Moreover, the combination of RT and surgery is known to worsen the quality of life more than single modalities of treatment [33, 34].

The relationship between RT‐related toxicities and fatigue showed a strong association, probably influenced both directly, through the elevation of pro‐inflammatory cytokines like IL‐6, and indirectly, via the impact of these toxicities on patient lifestyle, such as sleep disturbances from lower urinary tract symptoms (LUTS). Other studies have linked radiation‐induced toxicities with elevated fatigue levels, reinforcing the importance of managing these toxicities to mitigate fatigue [35, 36].

In this context, alcohol consumption also plays an important role in amplifying fatigue as it could significantly worsen radiotherapy‐induced tiredness, irritate the bladder, and disrupt sleep [37].

Our study utilized the Cancer Linear Analogue Scale for fatigue assessment, a validated [38, 39, 40, 41, 42] and reliable measure despite the existence of other fatigue scales [43, 44, 45, 46]. In Maxwell's 1978 study published in the British Journal of Clinical Pharmacology, the performance of the Visual Analogue Scale (VAS) was evaluated in a psychophysical classroom setting [38]. The VAS was found to be simple and acceptable, showing good internal consistency for within‐subject changes. Then, Gough et al. and Coates et al. in 1983 evaluated the Linear Analogue Self‐Assessment (LASA), demonstrating a strong correlation with multi‐item quality‐of‐life measures in patients with advanced cancer [47, 48]. The conclusion was that a single‐item LASA asking the direct question “How would you rate your quality of life today?” represents a valid and reliable indicator of the QoL of patients with cancer. CLAS is a similar tool expanded in three domains. Finally, Van Belle et al. in 2005 published a comparison between the FACT‐F and VAS1 (energy level), 2 (quality of life), and 3 (ability to perform daily activities), finding a good agreement with International Statistical Classification of Diseases and Related Health Problems (10th revision) (ICD‐10) criteria [49]. Even if in Van Belle et al. CLAS is named VAS, the three domains are similar, and the final consideration is that it can be used as a rapid, easy‐to‐use tool for fatigue assessment. Moreover, we tested this tool and published related results in several settings over the years [41, 42]. While the heterogeneity of fatigue scales in the literature may limit direct comparisons, our approach has provided a homogeneous and comprehensive assessment of fatigue over a significant period. In this context, the calculated standard deviation (SD) of CLAS values consistently ranged between 1.8 and 2.2 across all classes, as also reported in the Results section. The threshold selected to define a clinically meaningful variation in CLAS values (≥ 2), originally derived from prior literature [50], is therefore further supported by the magnitude of variability observed in the present dataset.

Notably, the nurses played an essential role: helping patients regularly to recognize this under‐reported symptom by asking simple and direct questions has led to a more reliable assessment rather than giving a written questionnaire potentially determining an inaccurate evaluation because of a misinterpretation of the addressed items. It is known that fatigue is a multifactorial symptom with a multidimensional aspect to be acknowledged. However, some scales are too lengthy and burdensome for patients with advanced cancer, with multiple items, long completion time, and more complex administration [51]. On the contrary, CLAS score is a simple and manageable scale for both patients and interviewers.

This research represents one of the largest studies focused on fatigue in PC patients, notable for its long‐term, prospective data collection on fatigue. A key strength of our study is the systematic evaluation of fatigue at multiple critical points in the patient journey, allowing for a detailed examination of RT‐related fatigue onset.

This study, while extensive, is not without limitations. Its retrospective design may introduce selection bias and limit the ability to establish causality between RT and fatigue onset. The use of a single fatigue assessment scale, though consistent, may not capture the multidimensional nature of fatigue experienced by PC patients. Additionally, the lack of detailed information on patients' lifestyle changes post‐treatment and the potential influence of psychological factors on fatigue were not comprehensively assessed. Moreover, follow‐up was limited to 12 months, but fatigue levels are slightly worsened in the last assessment, raising the hypothesis that fatigue may persist beyond the observed period. Defining a clear endpoint for fatigue remains challenging as long‐lasting fatigue has been reported even years after treatment and in the absence of disease recurrence [52]. Future studies employing a prospective, multidimensional approach to fatigue measurement, incorporating both physical and psychological factors, are warranted to further elucidate the complex interplay between RT and fatigue in this population.

## 5 Conclusion

In our cohort, primarily treated with modern RT techniques, approximately 30% of patients exhibited moderate to high fatigue levels before undergoing RT. Less than 20% reported new‐onset fatigue attributable to RT. Factors such as treatment‐related toxicities, hypofractionation, alcohol consumption, and ADT were significant contributors to fatigue development.

## Author Contributions

**Milena Ferro:** investigation, writing – original draft, visualization, data curation. **Savino Cilla:** data curation, formal analysis, writing – review and editing. **Gabriella Macchia:** conceptualization, methodology, writing – review and editing. **Giuseppe Limosani:** investigation. **Paolo Bonome:** investigation. **Gianpiero Mastrogiorgio:** investigation. **Vincenzo Picardi:** investigation. **Carmela Romano:** formal analysis. **Marica Ferro:** investigation. **Mariangela Boccardi:** investigation. **Donato Pezzulla:** investigation. **Milly Buwenge:** investigation. **Alessio G. Morganti:** conceptualization, methodology, supervision. **Francesco Deodato:** conceptualization, methodology, validation, formal analysis, supervision, resources, writing – review and editing.

## Funding

The authors have nothing to report.

## Ethics Statement

Patients signed an informed consent form before the use of their clinical data.

## Conflicts of Interest

The authors declare no conflicts of interest.

## Data Availability

The data that support the findings of this study are available from the corresponding author upon reasonable request.
